# Comparison of Six Sensor Fusion Algorithms with Electrogoniometer Estimation of Wrist Angle in Simulated Work Tasks

**DOI:** 10.3390/s24134173

**Published:** 2024-06-27

**Authors:** Arvin Razavi, Mikael Forsman, Farhad Abtahi

**Affiliations:** 1 Division of Ergonomics, School of Engineering Sciences in Chemistry, Biotechnology and Health, KTH Royal Institute of Technology, 141 57 Huddinge, Sweden; arvraz@kth.se (A.R.); miforsm@kth.se (M.F.); 2 Unit of Occupational Medicine, Institute of Environmental Medicine, Karolinska Institutet, 171 77 Stockholm, Sweden; 3 Department of Clinical Science, Intervention and Technology, Karolinska Institutet, 171 76 Stockholm, Sweden; 4 Department of Clinical Physiology, Karolinska University Hospital, 141 86 Huddinge, Sweden

**Keywords:** IMU, wrist absolute angle, IMU-based human motion capture, orientation filter, complementary filter, multiplicative Kalman filter

## Abstract

Hand-intensive work is strongly associated with work-related musculoskeletal disorders (WMSDs) of the hand/wrist and other upper body regions across diverse occupations, including office work, manufacturing, services, and healthcare. Addressing the prevalence of WMSDs requires reliable and practical exposure measurements. Traditional methods like electrogoniometry and optical motion capture, while reliable, are expensive and impractical for field use. In contrast, small inertial measurement units (IMUs) may provide a cost-effective, time-efficient, and user-friendly alternative for measuring hand/wrist posture during real work. This study compared six orientation algorithms for estimating wrist angles with an electrogoniometer, the current gold standard in field settings. Six participants performed five simulated hand-intensive work tasks (involving considerable wrist velocity and/or hand force) and one standardised hand movement. Three multiplicative Kalman filter algorithms with different smoothers and constraints showed the highest agreement with the goniometer. These algorithms exhibited median correlation coefficients of 0.75–0.78 for flexion/extension and 0.64 for radial/ulnar deviation across the six subjects and five tasks. They also ranked in the top three for the lowest mean absolute differences from the goniometer at the 10th, 50th, and 90th percentiles of wrist flexion/extension (9.3°, 2.9°, and 7.4°, respectively). Although the results of this study are not fully acceptable for practical field use, especially for some work tasks, they indicate that IMU-based wrist angle estimation may be useful in occupational risk assessments after further improvements.

## 1. Introduction

Several studies have reported a significant correlation between hand-intensive work and work-related musculoskeletal disorders (WMSD) affecting the hands, wrists, forearms, elbows, and shoulders, leading to pain and disability [1,2,3,4,5]. Hand/wrist WMSDs are common in manufacturing and service industries, healthcare, construction, and office work. Some important risk factors for these conditions include heavy manual handling, repeated movements, forceful exertions, prolonged nonneutral wrist postures, and vibrations [2,3,6,7]. The majority of these field studies have relied mainly on self-reports or observation-based exposure estimations. Notably, only a few studies have incorporated technical, quantitative data in examining the exposure–response relationships, particularly concerning wrist activities [4,5,8].

### 1.1. Challenges with Traditional Measurement Techniques

Traditional quantitative measurement techniques, such as electrogoniometry [9,10] are expensive, and the sensor may mechanically wear out in a few days. Optical motion capture systems are costly and have low usability for unsupervised use. In addition, optical motion capture is limited to the controlled laboratory environment, restricting its widespread application [11]. Therefore, compact and portable inertial measurement units (IMUs) are considered an excellent low-cost wearable option for measuring human motion, including arm, trunk, and neck kinematics [8]. IMUs track a rigid body’s 3D position and orientation time without external references and typically measure acceleration (including acceleration and gravity), angular velocity, and magnetic field using accelerometers, gyroscopes, and magnetometers [12]. However, ambient magnetic disturbances are common, while the fusion algorithms rely on a homogeneous magnetic field. Previous research has explored sensor fusion techniques where the magnetometer data has been excluded to mitigate this error. The validity has been shown to be high in cases where the magnetic field is not needed nor used, such as in upper arm inclination measurements [13], where the fusion of gyroscope and accelerometer data has significantly higher validity than when only accelerometers are used [14].

### 1.2. Evaluation of Sensor Fusion Techniques for Wrist Motion Measurement

The wrist velocity can be estimated using only the gyroscopes, with an appropriate validity compared to electrogoniometers. Manivasagam and Yang (2022) [15] evaluated a new simplified inertial sensor method using gyroscope signals against an electrogoniometer for measuring wrist flexion velocity. Additionally, Casson et al. (2016) [16] compared motion measurements using accelerometers and gyroscopes, contributing to understanding the differences between these sensors for estimating wrist motion. A field study by Fethke (2020) [17] utilised inertial measurement units (IMUs) for wrist velocity measurement during common agricultural activities. This study employed direct measurement methods to assess biomechanical factors. They used IMUs to measure wrist velocity but not wrist posture; their reasoning for excluding postures was that “Wrist posture could not be calculated because, unlike the trunk and upper arm, the orientation of the wrist joint with respect to gravity cannot be assumed”.

The IMU-based estimation of wrist posture, i.e., 3D orientation estimation, particularly in terms of absolute angles, presents significant challenges in biomechanics research. Efforts have been made to enhance this estimation by applying fusion algorithms, which aim to utilise the IMU signals optimally. These algorithms aim to approximate a golden standard typically derived from optical tracers or electrogoniometers. Notably, some experiments have excluded magnetometer signals, experimenting with both the presence and absence of these measurements to determine their impact on the accuracy of wrist posture estimation. Poitras’ review [18] analysed six studies involving a total of 51 participants and describes the research on wrist joint estimation as inconsistent. Considerable variability was found in the accuracy of these measurements, as indicated by the root mean square error (RMSE) and correlation coefficients. RMSE values varied significantly from 2.2 to 30°, and correlation coefficients ranged from 0.62 to 0.99, suggesting fair to good validity. The review noted poorer results for radial–ulnar deviation movements than flexion-extension movements.

Zhou et al. (2010) [19] used a Kalman filter for the accelerometer and gyroscope fusion and proposed a kinematic model based on premeasured lengths of the upper and lower arms to reduce the drift of the angular signal. They reported a very low drift of 0.005 ms^−1^ and an RMS position error of less than 9 mm during five daily activities [19], and that the measurement drift in segment orientation was dramatically reduced after a Kalman filter was applied to estimate inclinations using accelerations and turning rates from gyroscopes. Therefore, although validation studies have yielded promising outcomes, Fethke et al. (2020), as previously mentioned, did not find it feasible to estimate wrist angles using IMUs in work environments. The Xsens motion capturing system (Xsens Technology BV, Enschede, The Netherlands) has been used to measure wrist posture in at least one field study [20]. However, to the best of our knowledge, there is no publicly accessible validation study of Xsens’s wrist posture estimation algorithm.

In their 2023 study, Chen et al. [21] investigated six sensor fusion algorithms. The algorithms incorporated the Rauch–Tung–Striebel smoother (RTS), the Multiplicative Extended Kalman Filter (MEKF), and the Maximum a posteriori (MAP). These algorithms were applied alongside a linear acceleration kinematic constraint (acc) and an additional degree of freedom constraint (acc-dof). The researchers reported low root mean square errors (RMSEs) of less than 4.4° for wrist tracking across all algorithms compared to an optical motion tracking system. However, the tasks assessed might not have required significant manual effort and frequently involved nearly vertical hand postures. These are less representative of movements and postures typically associated with assembly work. This discrepancy underscores the need for further validation studies on wrist posture, specifically focusing on kinematics that mirrors real-life working postures and movements more closely.

### 1.3. Significance and Application of Accurate Wrist Motion Measurement

Risk assessment is a crucial tool in identifying risks and prioritising measures to tackle the widespread occurrence of WMSD among various occupational groups. There are recommended action limits for both objectively measured wrist posture and angular velocity, which, if exceeded, indicate an increased risk for MSDs. Although IMU-based human motion measurements are gaining popularity in recent ergonomic studies, in occupational settings, the upper body has been limited to the head, arms, and trunk, leaving the measurements of wrist velocity and posture to traditional measuring methods [8]. There is yet to be a universally accepted guideline for using IMU-based motion capture to estimate wrist kinematics.

Developing algorithms for estimating wrist velocity and posture is essential, particularly in the context of proactive risk assessment tools for manual handling, such as the Risk Management Assessment tool for Manual handling Proactively (RAMP). These algorithms are crucial in quantifying and assessing musculoskeletal disorder risk factors associated with manual handling tasks [22]. In addition, accurate estimation of wrist velocity is essential for understanding hand and wrist movements during various activities, which is vital for assessing the risk of developing musculoskeletal disorders such as carpal tunnel syndrome (CTS) [23]. Moreover, the use of wearable sensors and edge computing in ergonomic platforms and the incorporation of innovative tools for safe manual handling underscores the need for accurate algorithms to estimate wrist velocity and posture. These algorithms contribute to developing precise and personalised ergonomic assessments, thereby improving the identification and management of musculoskeletal disorder risks in manual handling activities [24].

### 1.4. Research Focus and Expected Outcomes

The research focused on assessing various sensor fusion algorithms utilising two IMUs for calculating absolute wrist angles. This study specifically compared the IMU-based estimations of flexion and deviation angles, derived through different algorithms, against the angles recorded by an electrogoniometer. The expected outcome of this investigation is to contribute to assessing the reproducibility of IMU-based sensor fusion algorithms’ performance across different occupational contexts and a range of work-related tasks.

## 2. Materials and Methods

### 2.1. Participants

The experiment involved six healthy adult participants, two women and four men. None of the participants had any complaints in the elbow, forearm, wrist, or hand in the three months preceding the measurement. Five participants were right-handed, and one was left-handed. Table 1 shows the characteristics of the participants.

### 2.2. Simulated Work Tasks

The tasks in the experiment were designed to replicate realistic work tasks, which may include risk factors for hand/wrist WSMD. These factors involve repetitive movements, forceful exertions, prolonged nonneutral wrist postures, and vibrations.

**Figure 1:**
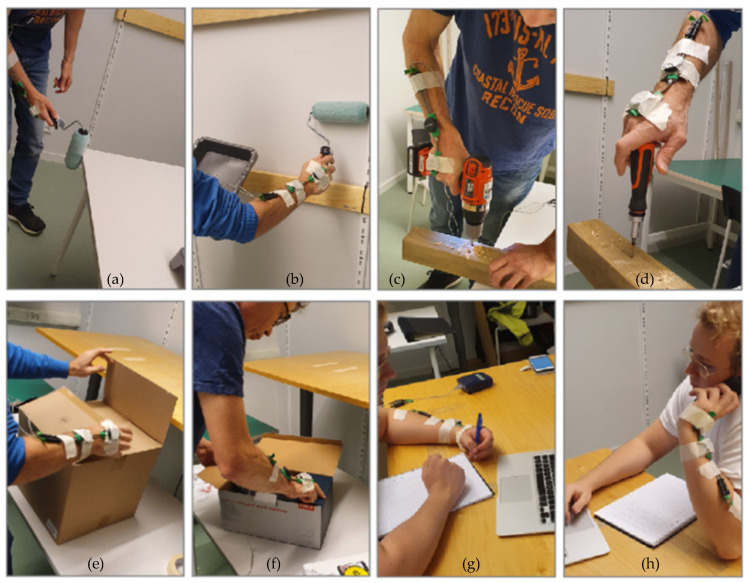
Painting task, the participants painted various vertical and horizontal surfaces (**a**,**b**), drilling and screwing with a screwdriver (**c**,**d**). Warehouse simulated task: boxes were picked up from the floor and placed on a shelf (**e**,**f**); simulated office work, including typing, taking notes, and making phone calls (**g**,**h**).

After a concise experiment introduction, electrogoniometer end blocks were affixed to the participant’s dominant hands and forearms, followed by the attachment of IMU sensors secured with additional tape. A reference neutral hand/forearm position (zero wrist flexion and deviation angle) was maintained for ten seconds at the start and end of each recording. During initial tasks commenced with controlled wrist movements, a metronome was utilised. The subsequent tasks were painting a surface with a paint roller, drilling holes in wood, screwing a screw into wood, lifting boxes from the floor to a shelf, and office/computer work, as shown in Figure 1:

- **Standard hand movement**: Flexion/extension and ulnar/radial deviation exercises were performed ten times each, first with the forearm on the table and then with the arm/forearm held vertically, at 30 bpm, 60 bpm, and 90 bpm using a digital metronome.
- **Painting**: The participants painted a table and a wall using a construction painter’s tasks. The task lasted 3–5 min.
- **Construction work**: To replicate the tasks of a worker exposed to hand-arm vibrations and fast hand/arm movements, the participants drilled holes in a hardwood plank, screwed with a manual screwdriver and removed screws, lasting 3–5 min.
- **Warehouse work**: Simulating warehouse work, the participants lifted, opened, and repacked boxes, placing them on a shelf, taking 2–4 min.
- **Office work**: This simulation involved a 10 min task of writing, reading, and answering a phone while sitting and standing behind a desk.

### 2.3. Instruments

A biaxial electrogoniometer (SG65 Biometrics Ltd., Newport, UK.), commonly employed in occupational exposure studies for measuring wrist flexion/extension and radial/ulnar deviation [4,5,9,25], was used as the reference standard for comparing the accuracy of the IMU-based angle estimation. The electrogoniometer output signal was calibrated and validated using a digital angle gauge and a robotic arm. This study utilised two IMU sensors (Movesense Ltd., Vantaa, Finland). These sensors were mounted on the electrogoniometers. The goniometer end blocks were securely attached to the dorsal surface of the wrist, with one end positioned over the third metacarpal and the other over the midline of the forearm. The threeaxis gyroscope, accelerometer, and magnetometer data from both IMU sensors were recorded at 52 Hz using the iOS mobile application Movesense Showcase v.1.0.5. The dual-axis signals of flexion/extension and radial/ulnar deviation from the goniometer were recorded at 128 Hz using the Mobi-8 logger (TMS International, Oldenzaal, The Netherlands). These signals were then stored for subsequent processing and analysis.

### 2.4. Orientation Filters

The rotational and translational movements of each IMU were recorded in three dimensions, capturing three-axis gyroscope, accelerometer, and magnetometer data. These individual signals were fused to calculate the orientation of each IMU relative to gravity and the earth’s magnetic field, which is usually expressed as a Eulerian angle and consists of three axes: angles, roll, yaw, and pitch [12,26]. Table 2 summarises the sensor fusion algorithms used in this study, ranging from simple first-order complementary filters to advanced Kalman filters. The rotational and translational movements of each IMU were recorded in three dimensions, capturing three-axis gyroscope, accelerometer, and magnetometer data. These individual signals were fused to calculate the orientation of each IMU relative to gravity and the earth’s magnetic field, which is usually expressed as a Eulerian angle and consists of three axes: angles, roll, yaw, and pitch [12,26]. The algorithms are briefly described in this section, and detailed information can be found in the reference for each algorithm. Furthermore, the implementation used in this study can be accessed from the supplementary GitHub repositories (https://github.com/uah-crablab/imu-constraint-matlab-el-wr) [21], accessed on 15 January 2024 and (https://github.com/how-chen/Biomech/tree/master/IMU/Inclination), accessed on 15 January 2024 [13].

#### 2.4.1. Multiplicative Kalman Filter with Linear Acceleration Constraint (mekf-acc)

The mekf-acc filter used in this experiment was implemented from a previous study by Chen et al. (2023) [21]. The algorithm integrates data from the Multiplicative Extended Kalman Filter (mekf) with linear acceleration measurements (acc), utilising gyroscopes and accelerometers to estimate joint angles. It considers linear acceleration constraints to enhance accuracy, assuming identical acceleration calculations at the joint centre from both sensors. For a detailed understanding of the algorithm and its mathematical derivation, please refer to Chen et al. (2023) [21].

Following experimental validation, the gyroscope noise was set to 0.01 and the constraint noise to 0.08.

#### 2.4.2. The Multiplicative Rauch–Tung–Striebel Smoother with Linear Acceleration Constraint (rts-acc)

The rts-acc filter enhances the multiplicative Kalman filter with a linear acceleration constraint, mek-acc, by incorporating a Rauch–Tung–Striebel (RTS) smoother. It refines state estimation by incorporating past and future IMU signal values through a two-pass Kalman filtering process, improving measurement accuracy. The details of the algorithm and the mathematical derivation are presented in the work of Chen et al. (2023) [21].

Following experimental validation, the gyroscope noise was set to 0.01 and the constraint noise to 0.05.

#### 2.4.3. Multiplicative Kalman Filter with Linear Acceleration and Dof Constraint (mekf-dof)

The mekf-dof filter is similar to mekf-acc, as previously described, but it includes the integration of a degree of freedom rotational constraint. This adjustment aims to limit wrist movement, thereby enhancing measurement accuracy. The details of the algorithm and the mathematical derivation are presented in the work of Chen et al. (2023) [21].

Following experimental validation, the gyroscope noise was set to 0.01, the linear constraint noise to 0.3, and the rotational constraint noise to 0.3.

#### 2.4.4. Multiplicative Kalman Smoother with Linear Acceleration and Dof Constraint (rts-dof)

The rts-dof filter combines the multiplicative Kalman filter with a Rauch–Tung–Striebel (RTS) smoother while also implementing a degree of freedom rotational constraint. This adjustment is aimed at limiting wrist movement to enhance the accuracy of measurements. The details of the algorithm and the mathematical derivation are presented in the work of Chen et al. (2023) [21].

Following experimental validation, the gyroscope noise was set to 0.01, the linear constraint noise to 0.3, and rotational constraint noise to 0.3.

#### 2.4.5. First-Order Complementary Filter (1st-comp)

The gyroscope and accelerometer complementary first-order filter used in this experiment was implemented from a previous study by Chen et al. (2018) [13]. This filter recursively calculates pitch and roll angles by integrating data from both gyroscopes and accelerometers. A crucial aspect of this process is the incorporation of a filter tuning parameter, denoted as β, which ranges between 0 and 1. A *β* value of 0 implies exclusive reliance on gyroscope-derived inclinations, while a value of 1 indicates sole dependence on accelerometer-derived inclinations. Following the observation of the various measurements, the tuning parameter for this filter, $\beta_{\theta }$ and $\beta_{\varphi }$ were selected to be 0.2. The details of the algorithm and the mathematical derivation are presented in the work of Chen et al. (2018) [13].

#### 2.4.6. Madgwick’s Second-Order Complementary Filter (2nd-comp)

Madgwick et al. (2011) [26] developed the second order filter algorithm used in this experiment. The details of the algorithm and the mathematical derivation are presented in the work of Madgwick et al. (2011) [26]. Resembling a second-order gradient descent complementary filter, this filter incorporates an accelerometer and a magnetometer in a Kalman-based approach to compute gyroscope bias drift over time. Following the observation of various measurements in this study, the tuning parameter *β* was decided to be 0.08.

### 2.5. Data Processing

The quaternion difference, ∆*q*, indicates the rotation between two quaternions and is calculated by multiplying the reference unit quaternion by the inverse of the second unit quaternion [27]. To determine the absolute wrist angle, the unit quaternion outputs from the forearm and hand orientation estimation sensor fusion algorithms were used. Equation (1) was employed to compute the quaternion difference between the forearm and hand unit quaternions.

(1) $$∆q=q_{forearm}⨂{q_{hand}}^{-1}$$

The quaternion difference was converted into Euler angles to extract flexion/extension and radial/ulnar deviation. Based on the orientation algorithm and the IMU sensor mounting direction, the roll (*X*-axis rotation) and pitch (*Y*-axis rotation) angles represent the intended wrist movements. To ensure uniformity in the experiment, all measurements were taken using the same IMU placement on the forearm and hand. The roll and pitch outputs for each orientation algorithm were hardcoded in the final script.

The measurement data from this study were processed and analysed using MATLAB 2021 (The MathWorks, Inc., Natick, MA, USA). The three-axis IMU signals of the forearm and hand, including the accelerometer, gyroscope, and magnetometer, were initially captured at 52 Hz. However, due to some inconsistencies in the recorded data, both signals were interpolated to match the shortest data length to compensate for the missing measurement points. The actual sampling frequencies were, nevertheless, very close to 52 Hz. The IMU signals were then resampled to 40 Hz. To eliminate artefacts unrelated to human motion, a Butterworth fourth-order low-pass filter with a cutoff frequency of 5 Hz was applied. This filtering approach is supported by the findings of Hansson et al. (1996), who demonstrated that 99.5% of the signal power in occupational repetitive tasks lies within the 0–5 Hz frequency band [9]. The three-axis forearm and hand data were converted to a unit quaternion in the global coordinate system using orientation estimation filters. The angle between the two sensors was finally calculated by multiplying the forearm’s quaternion by the inverse quaternion of the hand (Equation (1)).

The goniometer signal was resampled to 40 Hz to match the frequency of the IMU data. To correct for the initial offset in wrist angle measurements, the average signal value between seconds 2 and 6 was computed and subtracted from each respective signal. MATLAB’s ‘finddelay’ function was employed to synchronise the estimated IMU wrist angle signal with the goniometer data by identifying the temporal lag between the two signals. Subsequently, both signals were trimmed to equal lengths to ensure consistency in subsequent analyses. The synchronised signals were then assessed for cross-correlation agreement, and each signal’s 10th, 50th, and 90th percentiles were extracted for further evaluation.

### 2.6. Statistical Analysis

MATLAB 2021 was used to analyse the synchronised processed signals from various IMU wrist angle estimation algorithms and the reference wrist angle signal from the electrogoniometer for each experiment.

The cross-correlation coefficient between two signals, ranging from −1 to 1, was determined using MATLAB’s ‘xcorr’ function. A coefficient of 1 indicates perfect agreement between the signals, whereas a coefficient of 0 indicates no agreement.

Median values and the non-parametric Kruskal–Wallis test were employed (the Shapiro–Wilk test was used to assess the normality of the data). However, due to the small sample size, the results of the Kruskal–Wallis post-hoc analysis between tasks and methods are not reported in detail.

In ergonomics research, both in experimental and field studies, percentiles are typically used to describe the distribution function of wrist postures (see e.g., [4,9,10,25]). Both signals’ 10th, 50th, and 90th percentiles were extracted, and the percentile differences between the electrogoniometer and the IMU-based wrist angle estimation were computed.

## 3. Results

This section outlines the results of five simulated tasks performed by six participants, generating a dataset with 180 records assessed using six orientation filters.

### 3.1. Signal Processing

**Figure 2:**
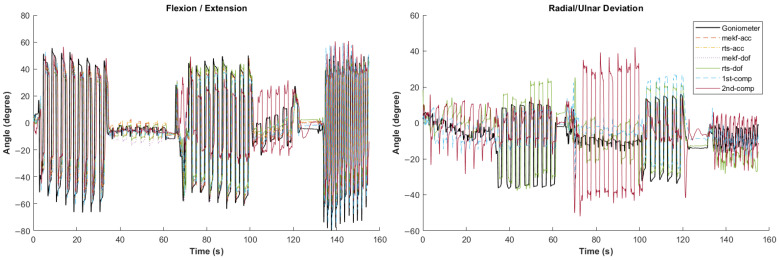
Synchronised wrist angle signal outputs from an inertial measurement unit (IMU) and an electrogoniometer illustrating the standard movement executed by Participant 4. Data is presented for six different orientation filter algorithms.

**Figure 3:**
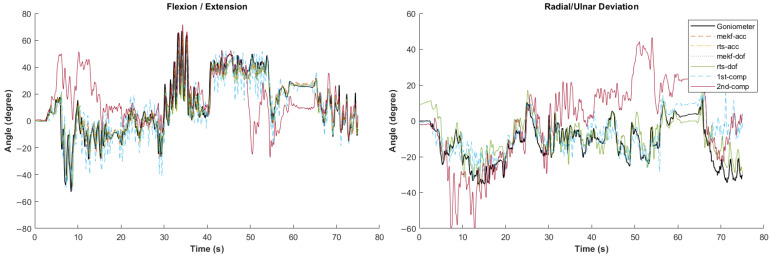
Synchronised wrist angle signal from IMU and electrogoniometer for the painting simulated task completed by Participant 6 using six different orientation filter algorithms.

**Figure 4:**
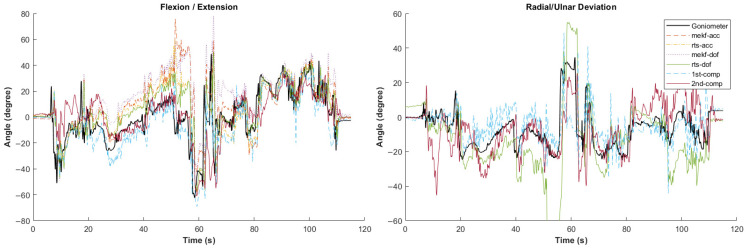
Synchronised wrist angle signal from IMU and electrogoniometer for the construction work simulated task completed by Participant 5 using six different orientation filter algorithms.

**Figure 5:**
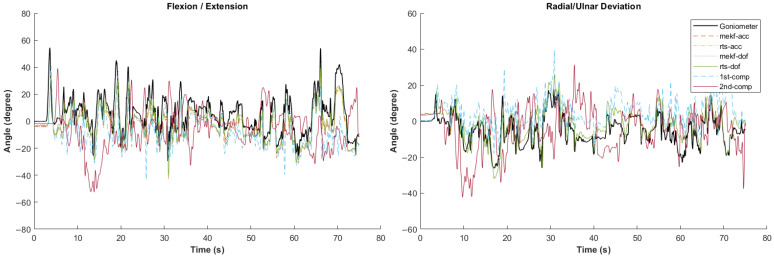
Synchronised wrist angle signal from IMU and electrogoniometer for the warehouse work simulated task completed by Participant 4 using six different orientation filter algorithms.

**Figure 6:**
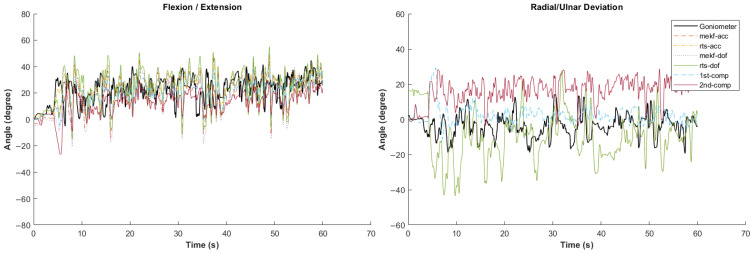
Synchronised wrist angle signal from IMU and electrogoniometer for the office work simulated task completed by Participant 6 using six different orientation filter algorithms.

Figure 2, Figure 3, Figure 4, Figure 5 and Figure 6 showcase selected sample signal plots from each simulated task performed by one participant. The reference signals from the electrogoniometer are represented in black, while the IMU-based wrist angle estimations obtained from each orientation filter are depicted in colours. These plots offer a visual representation of wrist movements performed during the tasks.

### 3.2. Correlation between the IMU and the Electrogoniometer-Based Signals

The correlation coefficient, ranging from −1 to 1, measures the similarity between two signals. A coefficient of 0 signifies no similarity, while 1 denotes complete similarity. Table 3 displays the median, mean and standard deviation of correlation coefficients for flexion/extension and radial/ulnar deviation across all five simulated tasks, categorised by the orientation algorithm. Considering the median across tasks and algorithms, the correlation coefficient was 0.71 for flexion/extension and 0.53 for radial/ulnar deviation. The algorithms mekf-acc, rts-acc, and rts-dof emerged as the top performers in this experiment, demonstrating comparable effectiveness. They achieved median correlation coefficients of 0.75–0.78 for flexion/extension and 0.64 for radial/ulnar deviation. However, these algorithms showed the poorest performance in construction work applications.

**Figure 7:**
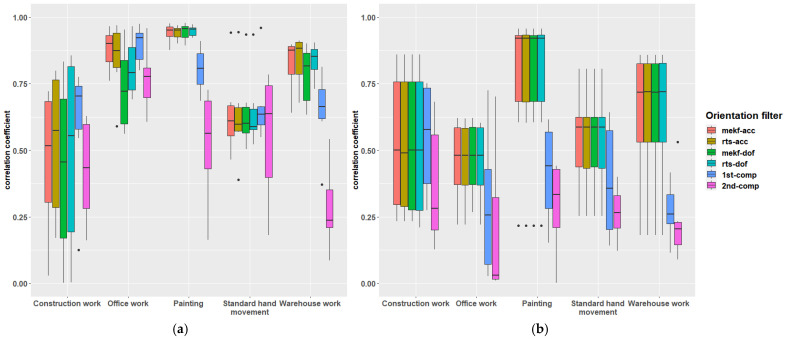
Boxplots of the correlation coefficients between the IMU-based wrist angle estimation and the electrogoniometer divided by different simulated work tasks for every orientation algorithm, (**a**) flexion/extension, (**b**) radial/ulnar deviation. Boxplots are created in R Studio version 1.4.1717. Dots represent outliers.

Figure 7 visually summarises the data presented in Table 3 in a boxplot where each orientation algorithm’s mean correlation coefficient is broken down into its corresponding simulated work task. According to the plot, the simulated work task with the best wrist flexion/extension estimation performance was the painting job, followed by office work using the mekf-acc and rts-dof, respectively. As can be understood from the boxplots and verified by the Shapiro–Wilk Test, data are likely not normally distributed. The Kruskal–Wallis test support the presented results based on the median (i.e., that the coefficients of the methods are different from each other). Notably, most algorithms exhibit lower performance in estimating radial/ulnar deviation.

### 3.3. The Discrepancy in Wrist Angle Estimation by Using IMUs and Electrogoniometer

The mean absolute differences (errors), in three percentiles, between the reference signal from the electrogoniometer and the IMU-based wrist angle estimations are presented in Table 4 and Table 5. Across all combinations of simulated work tasks and orientation algorithms, the mean absolute errors for the 10th, 50th, and 90th percentiles were as follows: for flexion/extension, they were 9.9°, 4.2°, and 8.8°, respectively; for radial/ulnar deviation, the errors were 12.2°, 6.3°, and 12.4°, respectively.

The mekf-acc, rts-acc, and rts-dof from 2.4.1 were once again the best-performing algorithms, while no orientation algorithm consistently outperformed the others with the lowest mean absolute error of 9.3°, 2.9° and 7.4° for the 10th, 50th, and 90th percentiles of wrist flexion/extension, respectively.

Variations in performance among different orientation algorithms were observed across the 10th, 50th, and 90th percentiles of radial/ulnar deviation. Nevertheless, the same algorithms—mekf-acc, rts-acc, and rts-dof—emerged as leaders.

## 4. Discussion

In this study, the similarity between IMU-based wrist angle estimation for flexion/extension and radial/ulnar deviation was evaluated by comparison to a biaxial electrogoniometer, which is considered the gold standard method for ergonomic assessments of wrist posture in field studies [4,9,10]. Six participants conducted one standardised movement and four simulated work tasks, which were analysed using six different orientation algorithms. These measurements produced correlation coefficients, median values for the algorithms, with electrogoniometer signals for flexion/extension and radial/ulnar deviation, ranging from 0.53 to 0.78 and 0.22 to 0.64, respectively. The mean absolute errors for the best-performing algorithm at the 10th, 50th, and 90th percentiles for flexion/extension were 9.4, 2.9, and 10.3 degrees, respectively. The errors for radial/ulnar deviation were 13.88, 6.35, and 13.9 degrees at the 10th, 50th, and 90th percentiles, respectively.

### 4.1. Comparison to Previous Research

The findings of this study align with those reported in the literature and summarised by Poitras et al. in their review [18]. Their review included six studies encompassing a total of 51 participants. The correlation coefficients between IMU measurements and an optical gold standard ranged from 0.62 to 0.99 for flexion/extension and radial/ulnar deviation, respectively. Additionally, the root-mean-square error varied across studies, ranging from 2.2 to 30 degrees for flexion/extension and from 3 to 30 degrees for radial/ulnar deviation [18].

Chen et al. (2023) [21] reported an error of 1.5–2.2 degrees for their best-performing algorithm (utilising gyroscope and accelerometer data) in estimating wrist joint angles of 13 participants engaged in a simple material transfer task lasting over 30 min. This error magnitude, notably lower than the error observed with the same algorithms in the current study, may be attributed to the lower complexity of tasks and the absence of high-velocity wrist movements during the material handling task. The reduced RMSE compared to our study may also be explained by the differing capabilities and sensitivities of the gold standards employed. Optical systems, known for their high spatial accuracy in capturing complex movements, could provide more precise reference data than electrogoniometers, which, despite their accuracy, may have limitations such as susceptibility to alignment errors. Consequently, the choice of gold standard—optical systems versus electrogoniometers—could significantly contribute to the differences in reported accuracy of IMU-based wrist angle measurements.

### 4.2. Variation between the Simulated Work Tasks

Figure 7 reveals varying correlation coefficients between the IMU-based filters and the electrogoniometer across different tasks. The discrepancies among task types typically exceed those among the filters. Since the data is not normally distributed, these observations were also checked using a non-parametric statistical method. When the Kruskal–Wallis test was employed to verify the drawn conclusion, it did ensure that the observed differences were statistically significant. Therefore, if an IMU-based filter is planned for use in a study involving one or more specific work tasks, it should be verified (either through a validation phase or based on previous studies) that the filter performs well in the included tasks. Before IMUs can be widely used in field studies, there is a need for substantial validation in different work tasks, alongside further improvements to the filters. Hence, more studies similar to this one are needed, preferably including both IMUs and electrogoniometers, as well as optical motion capture systems, in the same trials.

### 4.3. Variation between the Orientation Algorithms

In this study, we employed two groups of orientation filters: relatively simple complementary filters (first and second order; 1st-comp and 2nd-comp), which calculate joint angles based on the inclination difference between two IMUs placed on body segments on both sides of the joint, and four advanced multiplicative Kalman filters with linear and/or rotational motion constraints: mekf-acc, rts-acc, mekf-dof, and rts-dof. When using linear motion constraints, it is assumed that the linear properties (acceleration, velocity, or position) at the joint centre are uniform between the two IMUs on adjacent segments. In contrast, a rotational constraint limits the rotational motion of joints within one of the three motion planes or according to their range of motion [21]. Orientation filters with linear and/or rotational motion constraints are more computationally intensive and are generally unsuitable for real-time measurement analyses due to their extensive computational requirements and the need for complete signals from both segments to calculate alignment parameters. Complementary filters are quicker to execute and can be utilised for interactive result presentations.

Figure 7 and Table 3 show that the four orientation filters with linear and/or rotational motion constraints demonstrate a higher mean correlation between wrist flexion/extension measurements obtained from the electrogoniometer and IMU. However, these correlation factors are insufficient to replace goniometers with IMUs in field studies. More research is needed to improve the estimation based on IMU measurements and identify specific work task types where IMU-based wrist angle estimation may be valid. One such task could be the manual handling task simulated by Chen et al. [21]. Initially, it is necessary to improve and validate the best IMU-based filters. Subsequently, it should be investigated whether rapid complementary filters can be improved to the point where they may be used for sufficiently valid interactive result presentations.

A study by Nunes et al. [28] focused on postural wrist risk assessment in an automotive assembly line using IMUs. The research involved a comparative study with data collected in both laboratory and assembly line settings. However, the study did not compare the IMU-based posture estimation with a gold standard measurement, such as optical motion capture or electrogoniometers. An interesting finding was the difference between the left and right wrist for the intraclass correlation coefficient (ICC) using two different IMU systems. The lower ICC for the right wrist could be related to the faster movement of the dominant wrist compared to the non-dominant one.

A visual analysis of the results from the various orientation algorithms used in each simulated work task suggested that vibration and rapid hand movements may contribute to a drift in the IMU-based wrist angle estimation relative to the drift-free electrogoniometer reference signal. The choice of orientation algorithm is a significant factor in the error level observed in our experiment, making it a critical consideration for accurate measurements.

### 4.4. Use of Technical Measurements in Ergonomic Risk Assessment Methods

Common ergonomic risk assessment methods, such as Rapid Upper Limb Assessment (RULA) [29] and the European Assembly Worksheet (EAWS) [30], primarily focus on the assessment of individual tasks through expert observation, typically conducted over short periods in real-world settings or via offline video analysis. The integration of technical measurements, such as Inertial Measurement Unit (IMU)-based posture and movement monitoring, should be considered in the refined version of these tools, emphasising the importance of measurement validity and user experience design to enhance task awareness. Table 6 summarises the RULA, and Risk Management Assessment Tool for Manual Handling Proactively (RAMP) [22] criteria for wrist posture.

Investigating the efficacy of criteria established for observational assessments versus the necessity to adapt these methods for longer technical measurements could yield significant long-term benefits. For example, the RAMP approach considers the percentage of working time in specific postures, which may be more pertinent for translating technical measurements over a full working day. A simpler approach, focusing on average load (50th percentile), top load (90th percentile), and minimum load (10th percentile), could streamline the use of technical measurements. This simplification would provide ergonomists, industrial designers, and workers with valuable tools for ergonomic risk assessment, designing new workstations and tasks, and improving work techniques. Such advancements would ultimately contribute to a comprehensive ergonomic assessment framework, integrating real-time data and user-centred design for improved occupational health and safety.

Future work should evaluate the impact of estimation errors at the limb level on ergonomic risk assessment tools, especially in scenarios where scores approach threshold values. Understanding the potential consequences of inaccuracies in capturing limb postures and movements is crucial. These errors could lead to misclassifications of risk levels, emphasising the necessity for further research into enhancing the precision of ergonomic assessment tools.

### 4.5. Limitations of the Study

Using a custom-cut hook and loop tape, the IMU sensor was mounted to the electrogoniometer. While this method may have introduced errors in the IMU sensor readings, we consider these errors negligible.

To further evaluate the performance of the orientation algorithm used in this study, performing the same simulated work tasks with an electrogoniometer and an optical motion capture system as the gold standard for measurement would be appropriate. The use of an electrogoniometer for our field studies was chosen due to its practicality and established reliability in ergonomic assessments. Electro-goniometers are used in field settings for their ease of use and ability to provide consistent measurements in various environments. An important advantage of optical motion capture is its three-dimensional coordinate system, which more closely aligns with the output from IMU-based wrist angle estimations. We plan to incorporate optical motion capture and goniometers simultaneously in subsequent studies to enhance the robustness and accuracy of our findings, providing a more thorough validation of the IMU-based wrist angle estimation methods. This approach will ensure a comprehensive evaluation, leveraging the strengths of both measurement systems to improve the reliability and applicability of our algorithms in diverse settings.

While our study focused on simulating realistic hand-intensive activities, transitioning to real industrial environments for future evaluations is essential to capture the full complexity of actual settings. This shift will validate the practicality and robustness of the orientation algorithm under true working conditions, providing crucial insights into its performance among machinery vibration, operator variability, and environmental influences. The complex and evolving nature of industrial settings can reveal unique challenges and advantages of the algorithm that are not apparent in controlled environments. Real-world testing will ensure the accuracy and reliability of IMU-based wrist angle estimation, enhancing its performance and paving the way for successful integration into practical applications, ultimately improving ergonomics and worker safety in industrial contexts.

The primary aim of our study was to assess the accuracy of various algorithms across a range of work tasks, considering anatomical and behavioural differences among participants. For this purpose, our participant pool of six individuals was sufficient to support our results and highlight the need for further algorithm improvements before moving to real settings and investing resources in larger studies. However, we acknowledge that a larger and more diverse group would enhance the study’s validity and generalizability. Future research should involve a more extensive and varied population and be conducted in actual industrial settings rather than simulations. This approach will ensure the algorithms are reliable and effective across different populations and scenarios, ultimately supporting more accurate ergonomic risk assessments in practical field conditions.

## 5. Conclusions

This study assessed various orientation algorithms for estimating absolute wrist angles by comparing results from two IMU sensors against measurements from an electrogoniometer, considered the gold standard, under conditions designed to simulate real-world work environments. The correlation coefficients for these measurements varied between the algorithms, with flexion/extension ranging from 0.53 to 0.78 and radial/ulnar deviations between 0.22 and 0.64. Three multiplicative Kalman filter algorithms with different smoothers and constraints showed the highest agreement with the goniometer. These algorithms exhibited median correlation coefficients of 0.75–0.78 for flexion/extension and 0.64 for radial/ulnar deviation across the six subjects and five tasks.

The algorithm demonstrating the best performance exhibited mean absolute errors at the 10th, 50th, and 90th percentiles for flexion/extension of 9.4, 2.9, and 10.3 degrees, respectively, and for radial/ulnar deviations, the errors were 13.88, 6.35, and 13.9 degrees.

The results suggest that advancements in orientation algorithms are needed, likely through refining these algorithms further, adding additional constraints, or mitigating vibration noise artefacts. The current findings do not fully meet the standards of validity required for ergonomic field studies in wrist posture analysis, particularly for the top workload represented by the 10th and 90th percentiles, which exhibited errors exceeding 10 degrees. However, although the results of this study are not fully acceptable for practical field use, especially for certain work tasks, they indicate that IMU-based wrist angle estimation may become useful in occupational risk assessments with further improvements. Therefore, further improvements and rigorous validation of IMU-based wrist angle estimations in authentic work tasks remain critical for future research.

## Figures and Tables

**Table 1:** Participant details and body measurements.

Gender (M/F)	Handedness (Right/Left)	Age Mean (SD)	Height [cm] Mean (SD)	Weight [kg] Mean (SD)
4/2	5/1	43 (11.6)	173 (8.1)	77 (13.9)

**Table 2:** Sensor fusion algorithm details.

Abbreviation	Algorithm Name	Included Sensors	Method Type	Implementation	Method Settings
mekf-acc	Multiplicative Kalman filter with linear acceleration constraint	Gyroscope, accelerometer	Kalman	Chen et al. (2023) [21]	gyroscope noise = 0.01, constraint noise = 0.08
rts-acc	Multiplicative Rauch–Tung–Striebel smoother with linear acceleration constraint	Gyroscope, accelerometer	Rauch–Tung–Striebel smoother	Chen et al. (2023) [21]	gyroscope noise = 0.01, constraint noise = 0.05
mekf-dof	Multiplicative Kalman filter with linear acceleration and dof constraint	Gyroscope, accelerometer	Kalman	Chen et al. (2023) [21]	gyroscope noise = 0.01, linear constraint noise = 0.3, rotational constraint noise = 0.3
rts-dof	Multiplicative Kalman smoother with linear acceleration and dof constraint	Gyroscope, accelerometer	Rauch–Tung–Striebel smoother	Chen et al. (2023) [21]	gyroscope noise = 0.01, linear constraint noise = 0.3, rotational constraint noise = 0.3
1st-comp	First-order complementary filter	Gyroscope, accelerometer	Complementary	Chen et al. (2018) [13]	$\beta_{\theta }$ and $\beta_{\varphi }$ were selected to be 0.2.
2nd-comp	Madgwick’s second-order complementary	Gyroscope, accelerometer, magnetometer	Complementary	Madgwick et al. (2011) [26]	β=0.35

**Table 3:** Median of the correlation coefficient between IMU-based wrist estimation signal and electrogoniometer signal for flexion/extension and radial/ulnar deviation from various simulated work tasks using different orientation algorithms. Bold values in the Total row and column show the aggregated values for tasks and/or algorithms.

Orientation Algorithm/ Simulated Work Tasks	mekf-acc		rts-acc		mekf-dof		rts-dof		1st-comp		2nd-comp		Total	
	Flexion Extension	Radial Ulnar Deviation	Flexion Extension	Radial Ulnar Deviation	Flexion Extension	Radial Ulnar Deviation	Flexion Extension	Radial Ulnar Deviation	Flexion Extension	Radial Ulnar Deviation	Flexion Extension	Radial Ulnar Deviation	Flexion Extension	Radial Ulnar Deviation
Construction work	0.52	0.50	0.57	0.49	0.46	0.5	0.56	0.5]	0.7	0.58	0.43	0.28	**0.54**	**0.47**
Office work	0.9	0.48	0.87	0.48	0.72	0.48	0.79	0.48	0.92	0.26	0.78	0.03	**0.83**	**0.37**
Painting	0.95	0.92	0.95	0.92	0.96	0.92	0.95	0.92	0.81	0.44	0.56	0.33	**0.86**	**0.74**
Standard hand movement	0.61	0.59	0.6	0.59	0.6	0.59	0.59	0.59	0.64	0.36	0.64	0.26	**0.61**	**0.49**
Warehouse work	0.88	0.72	0.88	0.72	0.82	0.72	0.85	0.72	0.66	0.26	0.24	0.2	**0.72**	**0.56**
**Total**	**0.77**	**0.64**	**0.78**	**0.64**	**0.71**	**0.64**	**0.75**	**0.64**	**0.75**	**0.38**	**0.53**	**0.22**	**0.71**	**0.53**

**Table 4:** Mean absolute errors and standard deviation (SD) in degrees for the 10th, 50th, and 90th percentiles of flexion/extension from IMU-based wrist estimation against the corresponding percentiles from the reference measurement device, an electrogoniometer. Bold values in the Total row and column show the aggregated values for tasks and/or algorithms.

Orientation Algorithm/ Simulated Work Tasks	mekf-acc			rts-acc			mekf-dof			rts-dof			1st-comp			2nd-comp			Total		
	10th _Percentile_	50th _Percentile_	90th _Percentile_	10th _Percentile_	50th _Percentile_	90th _Percentile_	10th _Percentile_	50th _Percentile_	90th _Percentile_	10th _Percentile_	50th _Percentile_	90th _Percentile_	10th _Percentile_	50th _Percentile_	90th _Percentile_	10th _Percentile_	50th _Percentile_	90th _Percentile_	10th _Percentile_	50th _Percentile_	90th _Percentile_
Construction work	28.71 (58.07)	6.94 (7.75)	13.54 (8.57)	28.68 (59.2)	4.37 (5.48)	28.63 (46.34)	37.67 (56.53)	6.69 (4.84)	13.29 (9.58)	39.38 (58.86)	3.11 (2.19)	7.38 (7.99)	5.04 (3.73)	3.64 (3.77)	12.45 (9.58)	8.65 (4.86)	4.99 (6.36)	8.94 (9.14)	**24.69 (46.05)**	**4.95 (5.18)**	**14.04 (20.34)**
Office work	4.68 (4.08)	2.29 (1.21)	5.05 (4.61)	4.85 (4.59)	3.05 (2.67)	4.08 (0.86)	5.19 (3.83)	6.34 (5.66)	10.72 (15.95)	7.83 (3.38)	7.31 (4.55)	11.32 (17.33)	5.88 (6.93)	5.45 (3.95)	7.2 (3.51)	8.22 (7.83)	11.22 (11.37)	11.97 (7.43)	**6.11 (5.18)**	**5.94 (6.19)**	**8.39 (10.10)**
Painting	2.65 (1.51)	2.27 (1.72)	3.80 (3.31)	3.36 (2.19)	3.40 (1.45)	3.50 (2.62)	2.77 (1.82)	2.64 (1.89)	3.49 (3.45)	2.97 (2.06)	3.26 (2.39)	4.00 (2.52)	7.66 (4.63)	4.83 (2.91)	9.81 (5.02)	4.00 (4.87)	4.96 (2.54)	8.36 (6.59)	**3.90 (3.41)**	**3.56 (2.29)**	**5.49 (4.67)**
Standard hand movement	5.85 (4.67)	1.90 (1.64)	9.36 (5.87)	7.09 (3.50)	0.91 (1.34)	9.28 (7.08)	8.95 (6.92)	2.70 (2.41)	7.60 (2.9)	8.77 (5.01)	1.10 (0.98)	8.67 (5.36)	5.04 (3.71)	1.25 (1.29)	6.60 (1.70)	18.69 (9.07)	1.85 (1.38)	11.42 (6.65)	**9.06 (7.08)**	**1.62 (1.58)**	**8.82 (5.15)**
Warehouse work	4.63 (1.56)	4.40 (2.78)	6.01 (4.35)	3.14 (1.65)	2.77 (2.35)	4.65 (4.05)	5.73 (4.61)	5.58 (6.46)	7.32 (7.92)	4.27 (3.13)	3.48 (4.36)	5.48 (5.49)	5.87 (3.31)	5.02 (4.30)	9.54 (5.58)	12.71 (6.56)	6.26 (7.78)	11.23 (8.09)	**6.06 (4.78)**	**4.58 (4.83)**	**7.37 (6.14)**
**Total**	**9.30 (26.22)**	**3.56 (4.08)**	**7.55 (6.34)**	**9.43 (26.63)**	**2.90 (3.06)**	**10.03 (21.84)**	**12.06 (27.20)**	**4.79 (4.65)**	**8.48 (9.24)**	**12.65 (28.21)**	**3.65 (3.61)**	**7.37 (8.99)**	**5.90 (4.43)**	**4.04 (3.53)**	**9.12 (5.71)**	**10.45 (8.11)**	**5.86 (7.12)**	**10.39 (7.24)**	**9.96 (22.22)**	**4.13 (4.58)**	**8.82 (11.22)**

**Table 5:** Mean absolute errors and standard deviation (SD) in degrees for the 10th, 50th, and 90th percentiles of radial/ulnar deviation from IMU-based wrist estimation against the corresponding percentiles from the reference measurement device, an electrogoniometer. Bold values in the Total row and column show the aggregated values for tasks and/or algorithms.

Orientation Algorithm/ Simulated Work Tasks	mekf-acc			rts-acc			mekf-dof			rts-dof			1st-comp			2nd-comp			Total		
	10th _Percentile_	50th _Percentile_	90th _Percentile_	10th _Percentile_	50th _Percentile_	90th _Percentile_	10th _Percentile_	50th _Percentile_	90th _Percentile_	10th _Percentile_	50th _Percentile_	90th _Percentile_	10th _Percentile_	50th _Percentile_	90th _Percentile_	10th _Percentile_	50th _Percentile_	90th _Percentile_	10th _Percentile_	50th _Percentile_	90th _Percentile_
Construction work	29.91 (39.63)	5.92 (6.26)	20.91 (43.42)	29.96 (39.64)	5.98 (6.19)	20.69 (43.53)	30.16 (39.5)	6.00 (6.17)	21.19 (43.28)	30.66 (39.28)	6.27 (5.96)	21.15 (43.3)	4.61 (4.52)	6.18 (6.24)	7.82 (8.84)	8.18 (6.68)	9.73 (9.48)	7.92 (5.7)	**22.25 (32.12)**	**6.68 (6.48)**	**16.61 (33.62)**
Office work	19.85 (6.97)	13.00 (6.83)	10.3 (9.46)	19.84 (6.97)	13.00 (6.83)	10.30 (9.45)	20.06 (6.87)	13.66 (6.00)	10.95 (10.31)	19.84 (6.96)	13.00 (6.83)	10.29 (9.46)	9.71 (5.94)	3.41 (1.15)	8.91 (7.64)	8.26 (5.43)	11.66 (9.60)	17.62 (12.66)	**16.26 (8.01)**	**11.29 (7.19)**	**11.4 (9.65)**
Painting	3.50 (3.38)	4.24 (5.40)	6.35 (4.48)	3.52 (3.40)	4.23 (5.41)	6.36 (4.50)	3.50 (3.38)	4.22 (5.41)	6.36 (4.49)	3.51 (3.38)	4.22 (5.41)	6.36 (4.48)	9.69 (6.16)	7.58 (3.92)	8.29 (3.42)	9.65 (10.46)	5.35 (4.36)	12.81 (8.30)	**5.56 (6.02)**	**4.97 (4.81)**	**7.75 (5.36)**
Standard hand movement	9.98 (18.79)	5.49 (3.30)	23.54 (45.26)	9.97 (18.79)	5.49 (3.30)	23.54 (45.26)	9.98 (18.79)	5.49 (3.30)	23.54 (45.26)	9.95 (18.74)	5.48 (3.28)	23.54 (45.26)	8.43 (6.16)	3.52 (3.74)	16.04 (7.71)	8.65 (6.37)	4.46 (3.91)	9.57 (5.34)	**9.49 (14.6)**	**4.99 (3.31)**	**19.96 (34.83)**
Warehouse work	6.08 (4.55)	3.04 (2.53)	4.54 (3.01)	6.09 (4.57)	3.04 (2.53)	4.54 (3.02)	6.08 (4.55)	3.04 (2.53)	4.54 (3.01)	6.08 (4.55)	3.04 (2.53)	4.54 (3.01)	9.61 (4.88)	4.92 (4.07)	10.36 (4.65)	11.6 (8.36)	5.16 (4.48)	10.35 (6.08)	**7.59 (5.50)**	**3.71 (3.13)**	**6.48 (4.61)**
**Total**	**13.86 (21.08)**	**6.34 (5.95)**	**13.13 (27.57)**	**13.88 (21.09)**	**6.35 (5.93)**	**13.09 (27.59)**	**13.96 (21.08)**	**6.48 (5.94)**	**13.32 (27.59)**	**14.01 (21.07)**	**6.40 (5.89)**	**13.18 (27.55)**	**8.41 (5.55)**	**5.12 (4.18)**	**10.28 (6.99)**	**9.27 (7.24)**	**7.27 (7.02)**	**11.65 (8.25)**	**12.23 (17.53)**	**6.33 (5.83)**	**12.44 (22.65)**

**Table 6:** Risk scores (or points) in three risk assessment tools for various angular ranges.

Wrist Joint Angle	RULA Score	RAMP Score
Wrist Flexion and Extension	1: Neutral (0–10°) 2: Bent (<15°) 3: Bent (>15°)	Extension of more than 40° or flexion of more than 30° 0: less than 30 min 1: 30 min and 1 h 2: 1–2 h 3: 2–3 h 5: 3–4 h 7: 4 h or more
Ulnar and radial deviation	Add +1 if the wrist is bent away from the midline	Deviation more than 10 ulnar or 15 radial 0: less than 30 min 1: 30 min and 1 h 2: 1–2 h 3: 2–3 h 5: 3–4 h 7: 4 h or more

## Data Availability

Data presented in the paper are available upon request.
